# Quantitative Image Analysis Reveals Distinct Structural Transitions during Aging in *Caenorhabditis elegans* Tissues

**DOI:** 10.1371/journal.pone.0002821

**Published:** 2008-07-30

**Authors:** Josiah Johnston, Wendy B. Iser, David K. Chow, Ilya G. Goldberg, Catherine A. Wolkow

**Affiliations:** 1 Laboratory of Genetics, NIA Intramural Research Program, National Institutes of Health, Baltimore, Maryland, United States of America; 2 Laboratory of Neurosciences, NIA Intramural Research Program, National Institutes of Health, Baltimore, Maryland, United States of America; University of Colorado, United States of America

## Abstract

Aging is associated with functional and structural declines in many body systems, even in the absence of underlying disease. In particular, skeletal muscles experience severe declines during aging, a phenomenon termed sarcopenia. Despite the high incidence and severity of sarcopenia, little is known about contributing factors and development. Many studies focus on functional aspects of aging-related tissue decline, while structural details remain understudied. Traditional approaches for quantifying structural changes have assessed individual markers at discrete intervals. Such approaches are inadequate for the complex changes associated with aging. An alternative is to consider changes in overall morphology rather than in specific markers. We have used this approach to quantitatively track tissue architecture during adulthood and aging in the *C. elegans* pharynx, the neuromuscular feeding organ. Using pattern recognition to analyze aged-grouped pharynx images, we identified discrete step-wise transitions between distinct morphologies. The morphology state transitions were maintained in mutants with pharynx neurotransmission defects, although the pace of the transitions was altered. Longitudinal measurements of pharynx function identified a predictive relationship between mid-life pharynx morphology and function at later ages. These studies demonstrate for the first time that adult tissues undergo distinct structural transitions reflecting postdevelopmental events. The processes that underlie these architectural changes may contribute to increased disease risk during aging, and may be targets for factors that alter the aging rate. This work further demonstrates that pattern analysis of an image series offers a novel and generally accessible approach for quantifying morphological changes and identifying structural biomarkers.

## Introduction

During development, tissues are built from newly synthesized components along synchronized morphogenic pathways. During aging, these tissues may deteriorate and exhibit functional declines. Aging-related structural and functional declines are particularly evidence in striated muscles, where loss of muscle mass and strength is referred to as sarcopenia. Although aging-related declines in muscle structure are universal and have been documented for invertebrate and vertebrate species, their causes remain elusive [1]. In addition, the relationship between structural and functional declines is not entirely clear. Muscle functional declines have been detected in the absence of obvious structural deterioration, suggesting that functional losses may precede structural declines [2]. However, the caveat exists that structural markers may not have been adequately sensitive to reveal subtle structural changes. To identify genes and pathways that maintain tissue structure and function in old age, new approaches for quantifying tissue morphology are needed. Such methodologies would also be useful for determining the extent to which adult tissues are structurally static or dynamic.

Morphological changes can be very complex and, therefore, difficult to quantify. Quantification of morphological change typically consists of assessing individual markers at discrete intervals. Studies to date have used manual approaches to visually assess differences between images of particular body features [1], [3], [4]. However, these studies are labor-intensive and, therefore, small in scale. Their sensitivity may be limited by cognitive processes and by visual perception [5]–[7]. In addition, scoring criteria are inherently subjective and may vary between participants and over time [4]. An alternative approach is to consider changes in overall structure rather than specific markers. An unbiased quantitative approach for morphological studies would provide novel objective measurements for experimental manipulations. For example, unbiased quantitative morphological assessments could be used to identify tissue morphologies that predict the rates of future functional declines during aging.

We have developed a computational model, based on pattern recognition, to quantitatively track complex morphological change through time. We used this model to investigate whether tissue structure was altered in a predictable fashion during adulthood and aging in the *Caenorhabditis elegans* pharynx. The *C. elegans* pharynx is a neuromuscular organ responsible for ingestion and mechanical disruption of bacterial food. *C. elegans* tissues are well-suited for structural studies as the organism is transparent and tissues are easily viewed by light microscopy. The pharynx is particularly useful for structure-function studies because pharynx function can be measured by its pump rate. During adult aging, the pharynx exhibits structural and functional declines, but the underlying causes remain obscure [8].

Applying the computational model to *C. elegans* pharynx images spanning young, middle and late adulthood revealed a characteristic pattern of morphology state transitions across this period. Thus, tissue morphology was not static in adult animals, but dynamic. Many genes are essential for pharynx biogenesis and function, but their effects on pharynx aging have not been examined. As an exploration of this area, we analyzed the effects of neurotransmission defects on pharynx morphological progression. Applying the model to two mutant strains with pharynx neurotransmission defects revealed changes in the timing of morphology state transitions, but not their overall pattern. This finding suggests that adult morphology state transitions occur independently of neurotransmitter availability in this tissue. Finally, we applied the model to a longitudinal design and identified a pattern of midlife pharynx morphologies that were correlated with the future rate of pharynx functional decline. This suggests that tissue function in old age is determined, in part, by changes occurring during mid-life. These studies support the existence of cellular processes that promote a dynamic, but characteristic, pattern of morphological changes in this tissue as it ages.

## Results

### Image pattern analysis over lifespan

**Figure 1:**
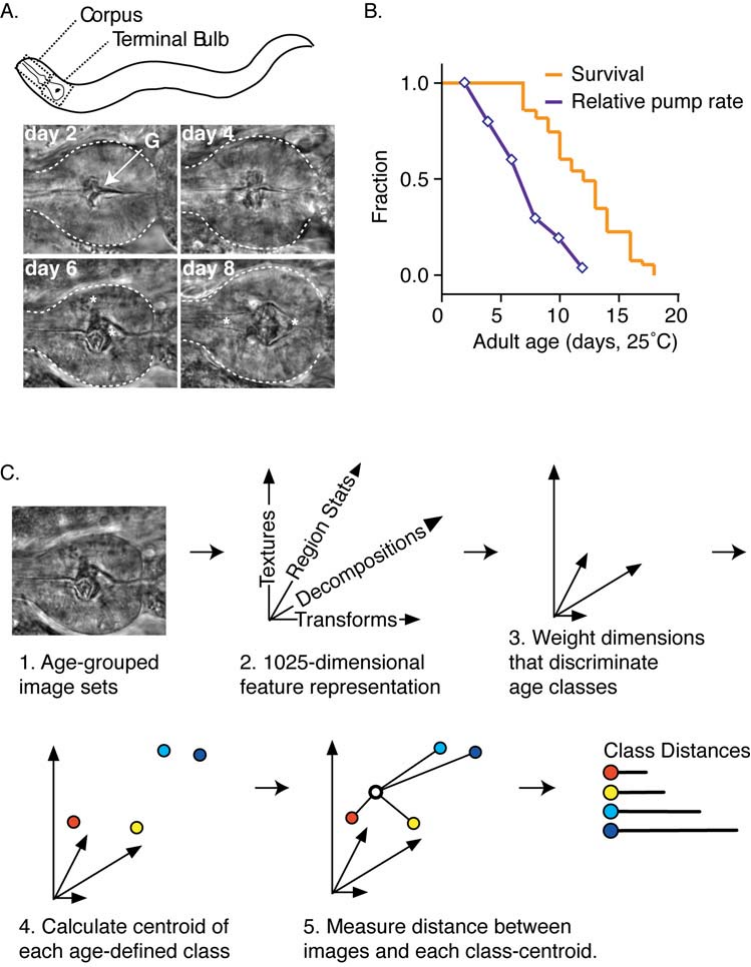
Overview of whole-image analysis. (A) Anatomical description of the *C. elegans* pharynx during adulthood. Cartoon illustrates the pharynx's relative position in the head, and representative images of the terminal bulb from four different ages are shown. Bacteria are ingested through the stoma, or mouth, and concentrated in the corpus region. Concentrated bacteria are passed to the terminal bulb (outlined with a dashed line for clarity) for mechanical disruption in the grinder (G) before transfer to the intestine. Structural aging is evident in the terminal bulb between adult days 2–8 as deterioration of the striated muscle fibers and disorganization in the grinder. (B) Declines in survival and pump rate occur during normal aging in *C. elegans*. Shown are representative curves for adult survival and pump rate (relative to day 2) in a population of *fem-1(hc17)* animals at 25°C. (C) General outline for the procedures used in pattern analysis of pharynx images (see text). 85 training images were used for each age group. The resulting class distances form the basis for similarity measurements and age-score predictions.

To characterize age-associated morphological changes in the pharynx, we used DIC microscopy to image the pharynx terminal bulb region from adult animals in early adulthood through old age (Fig. 1A). The *C. elegans* pharynx is a neuromuscular organ responsible for ingestion and mechanical disruption of bacterial food. This tissue is well-suited for studies of aging. Its morphology is easily assessed by light microscopy and pharynx function can be measured by its pumping rate. During adult aging, the pharynx exhibits structural and functional declines (Fig. 1A, B) [8]. To characterize age-associated morphological changes in the pharynx, we used differential interference contrast (DIC) microscopy to image the pharynx terminal bulb region from adult animals in early adulthood through mid-life and older. To facilitate production of age-synchronized populations, animals used in this study carried the *fem-1(hc17)* mutation blocking spermatogenesis [9]. Although the *fem-1(hc17)* animals are sterile, they exhibit lifespan characteristics similar to the wildtype. The sterile *fem-1(hc17)* animals were used as the baseline for “normal” aging throughout this study.

The same basic scheme was used for all image comparisons (Fig. 1C). First, images were randomly separated into training and test sets. The training images were divided into classes by age and used to build a computational model to study relationships among test images. The training images were divided into classes based on the animal's age at imaging (days 0, 2, 4, 6, 8, 10 and 12 of adulthood). A total of 85 training images representing all age groups were used to construct the model. The computational model was constructed by converting each training image into a point in a high-dimensional feature space, where each dimension corresponds to a different measurement of image content. These included texture statistics, polynomial decompositions, segmentation statistics, and image transforms [10], [11]. We used the Fisher Discriminant [12] to assign each feature dimension a weight based on its ability to distinguish age-defined classes and its insensitivity to variance in sample collection. The number of features used in the classifier was based on the Fisher scores of the image features. Initially, all features with Fisher scores 0.2 and above were used to train a classifier and test its performance. Subsequently, the feature selection threshold was increased, a new classifier trained with a smaller number of features, and its performance measured again. If the performance increased, the cycle would be repeated again. In most cases, the final classifier was based on the initial Fisher score selection criteria (≥0.2). This feature selection resulted in a Fisher weighted subspace containing 28 of the original set of 1025 features, which can also be used to represent each image as a single point. The set of points in the weighted subspace representing the training images for each class were then averaged to determine a centroid for each class. The final model consisted of feature weights and class centroids, and was built entirely without knowledge of the order of the age classes. The training images were used only to construct the model, and were not considered further in the analysis. The final model was used to evaluate 20 randomly-selected test images from each age by computing their selected image features, applying the corresponding weights, plotting the result in the weighted subspace, and measuring Euclidean distances to the model's class centroids.

**Figure 2:**
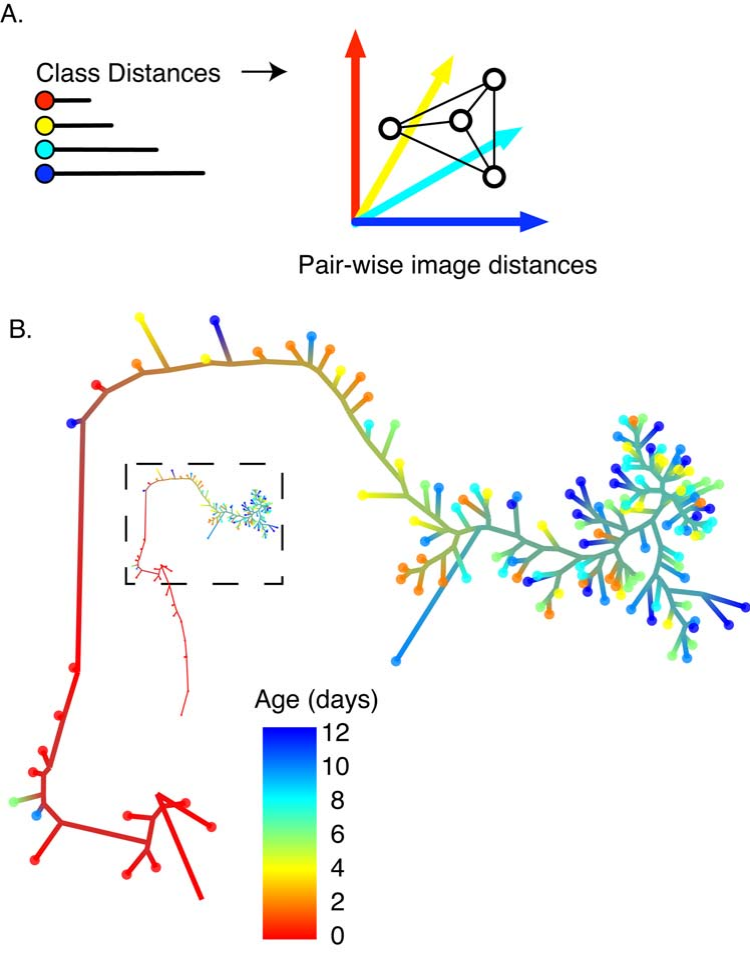
The aging model built on pharynx images from adult days 0–12 properly ordered the images by age. (A) Pair-wise image distances used to generate the dendrogram were calculated in the class-distance subspace, where each dimension is the distance from a test image to the centroid of an age-class. (B) Relationships between adult pharynx images from early to late life as determined by the aging model. Circles at branch end-points represent individual pharynx images, with color indicating the animal's age at the time of imaging. Branch lengths represent pair-wise distances between images, and branch colors represent the ages of proximal images. This diagram shows that images could be classified into categories corresponding to early adulthood (day 0, red), middle adulthood (days 2 and 4, orange) and late adulthood (day 6 and older, green and cyan). For training, 85 images from *fem-1(hc17)* adults aged day 0 to 12 were used for each class. The dendrogram was constructed from 20 test images selected at random from each class.

To compute similarities between test images, the distances between each image and each of the class centroids were used to form another subspace with a dimensionality equal to the number of classes (Fig. 2A). The positions of individual images within this class-distance subspace were plotted as individual points. The pair-wise distances between points, indicating similarity between images, were used to construct a dendrogram (Fig. 2B). During training, the model did not receive any information on the age-order of the training images. If the model were able to detect an age progression, then test images in the dendrogram would be ordered by their chronological age rather than clustered into unordered age groups. The dendrogram was correctly self-ordered with respect to age in many cases, particularly for the early age groups (days 0–4). In addition, this dendrogram portrayed a tight progression of morphologies during very early adulthood (days 0–2) (Fig. 2B, red & orange icons). This progression could reflect pharynx growth as part of the overall increase in body size during this time [13]. Images from animals older than day 4 were more closely spaced in the dendrogram than very young adults, suggesting greater image similarity after day 4. Later ages (days 10,12) showed an even greater degree of self-similarity, resulting in closer spacing in this visualization. Overall, this method defined a progression of image features that correlated with age, such that images from middle-aged animals (i.e. day 4, orange) were more similar overall to images from younger animals (day 0, red) than old adults (day 10, blue) and vice versa. It is also evident from this visualization that progressive changes in image features occur during this period of adulthood, thus this model can be used to assign a continuous age score for each individual pharynx image.

### Age-score predictions from image comparisons reveal distinct morphological states associated with aging

**Figure 3:**
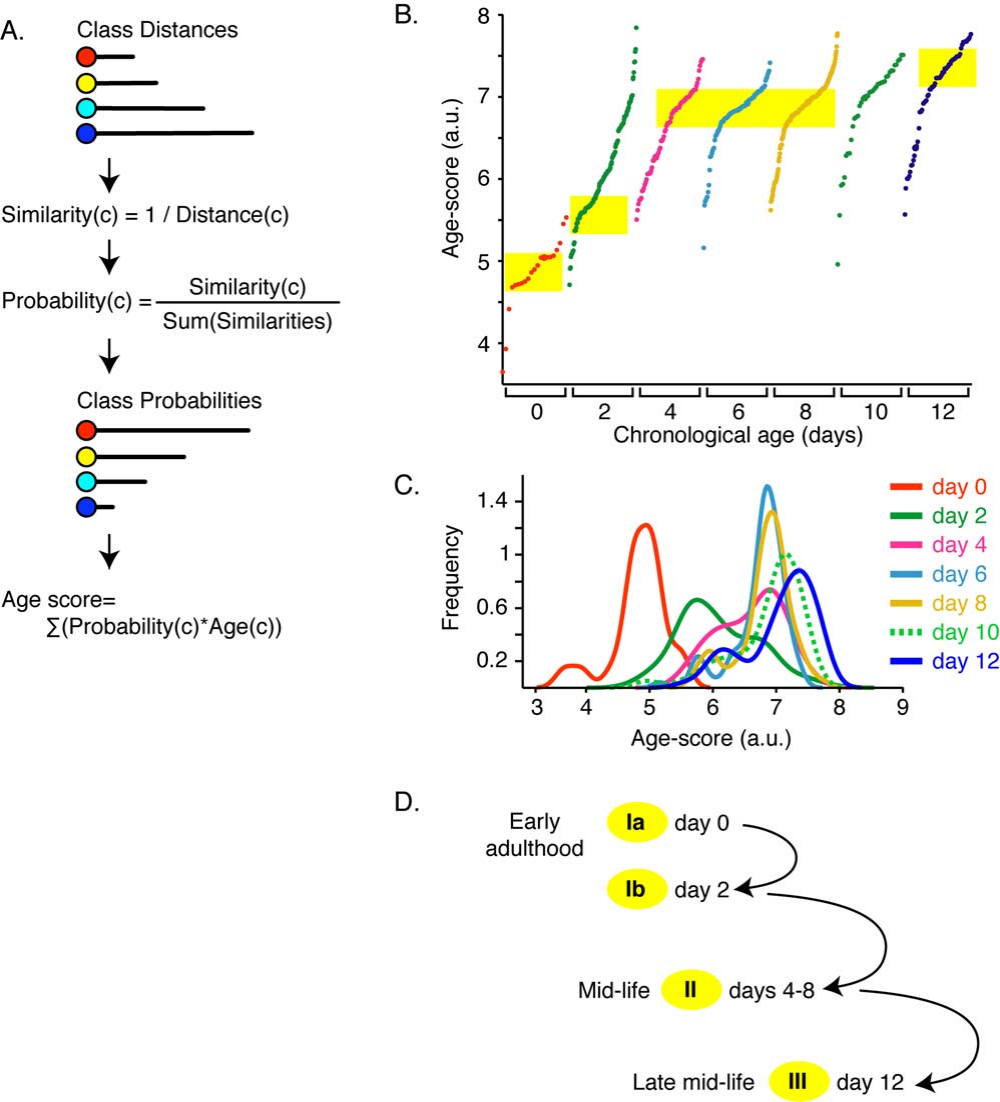
Age-scores for normal animals revealed distinct stages of adult morphology. (A) Age-scores were generated by converting each image's class distance into a class-probability, which were combined with class ages to produce an age-score. For training, 85 images from *fem-1(hc17)* adults aged day 0 to 12 were used for each class. (B) Age-scores for every test image from normal animals. The horizontal axis was ordered primarily by known age, and secondarily by age-scores. The yellow bars highlight inflections in the age-score distribution for images in each age group. These inflection regions constitute the stable morphology states depicted in (D). The number of test images for days 0–12 was 21, 143, 74, 91, 177, 40, and 62, respectively. (C) Probability distributions of age-scores, which can be interpreted as continuously sampled histograms with peaks indicating the most probable age-score. (D) Distinct stages of adulthood as determined by distribution of age-scores. Yellow ovals for each age state correspond to the inflections in the age-score distribution in part (B). See Table 1 and text for statistics.

For a more direct comparison between the test images, we calculated an age-score for each image from its distances to the class centroids (Fig. 3A, Table 1). For this analysis, a larger number of test images were used for each age group (day 0, 21 images; day 2, 143 images; day 4, 74 images; day 6, 91 images; day 8, 177 images; day 10, 40 images and day 12, 62 images). The Pearson's correlation coefficient between calculated age-score and chronological age was 0.54 (*p* = 5.34×10^−47^), indicating a statistically significant correlation between chronological age and the assigned age-score. Although most age groups were statistically distinguishable, age-scores overlapped between different age groups (Fig. 3B), but most age groups were statistically distinguishable (Table 1). Overall, these statistical tests indicated that there was a robust, but not perfect, correlation between the predicted age scores and actual age. This imperfect correlation likely reflects morphological heterogeneity of the population during aging. Consistent with increasing heterogeneity during aging, statistical significance was stronger in the younger age groups than among the older age groups. To simplify the representations of predicted age-score, probability density functions were generated for each age group (Fig. 3C). The peaks of these functions correspond to inflections in the rank-ordered age score predictions for each age group, indicating that morphologies occupied preferred states rather than being equally distributed within an age class. The first distinguishable states (Fig. 3D) occurred during early adulthood, at days 0 and 2 (age score, 4.5–5 and 5.5–6). These appeared to constitute subgroups of a single “young adult” state (Ia and Ib). The second major state occurred on days 4, 6 and 8 (age score, 6.5–7). We termed this state II and it appears to encompass mid-life. The third state (state III) represented morphologies associated with late life and included days 10 and 12. Although the day 10 population was not statistically distinguishable from days 6 and 8 (*p* = 0.47), the peak appeared slightly shifted from the overlapping peaks of days 6 and 8 (Fig. 3C). By day 12, the population was statistically distinguishable from state II, whether or not the calculation included day 10 data (*p* = 0.003 and *p* = 0.0025, respectively). This was termed state III and appeared to represent morphologies associated with late mid-life. We note that many images fell outside of the scores defining these morphology states, reflecting the heterogeneity in pharynx morphology at all ages.

**Table 1:** Statistical significance of predicted age-scores for each image age group.

	*p* *						
	day 0	day 2	day 4	day 6	day 8	day 10	day 12
day 0	0.5	<0.0001	<0.0001	<0.0001	<0.0001	<0.0001	<0.0001
day 2		0.5	<0.0001	<0.0001	<0.0001	<0.0001	<0.0001
day 4			0.5	0.08	0.0038	0.074	0.00024
day 6				0.5	0.12	0.29	0.0023
day 8					0.5	0.6	0.011
day 10						0.5	0.060
day 12							0.5

* *p*, one-tailed t-test comparing distribution of predicted age-scores pairwise between each image age group.

Independent analysis of a different pharynx region also identified similar morphology states representative of early-, mid- and later-adulthood. In the pharynx, the corpus muscles are located to the anterior of the terminal bulb muscles. Images of both the corpus and terminal bulb regions were collected from animals imaged on adult days 2, 4, 6 or 8. A model built using images of the corpus identified similar morphological states as for the terminal bulb, although with weaker statistics. Within this group of images, a Pearson correlation between predicted and chronological age for the corpus images was 0.47 (*p* = 6.46×10^−8^) compared to 0.68 (*p* = 2.17×10^−17^) for the terminal bulb. Interestingly, the morphological state of these two pharynx regions was synchronized in some animals. In particular, the shift between states I and II resulted in a low correlation between predicted and chronological age for corpus images from day 2 and 4 adults (0.17, *p* = 0.16), while within individual animals, the correlation between age scores for the corpus and terminal bulb was stronger and statistically significant (0.35, *p* = 0.0042).

### Comparison with manual analysis: Effect of slow pharynx pumping on tissue structure during aging

**Figure 4:**
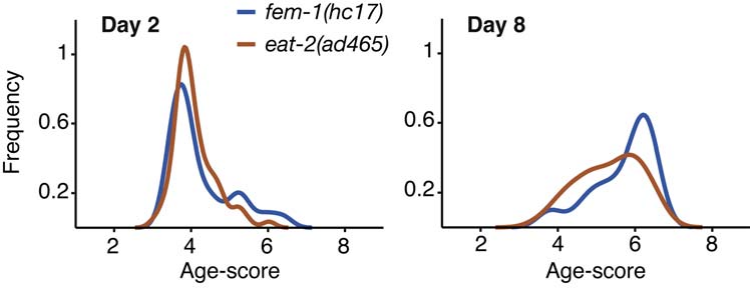
Probability density functions comparing morphological differences between normal and *eat-2(ad465)* slow-pumping animals at days 2 and 8. The model was trained on normal (*fem-1(hc17)*) animals at days 2 and 8. At day 2, *eat-2* mutants were indistinguishable from normal animals, while slightly lower age scores were evident on day 8.

**Figure 5:**
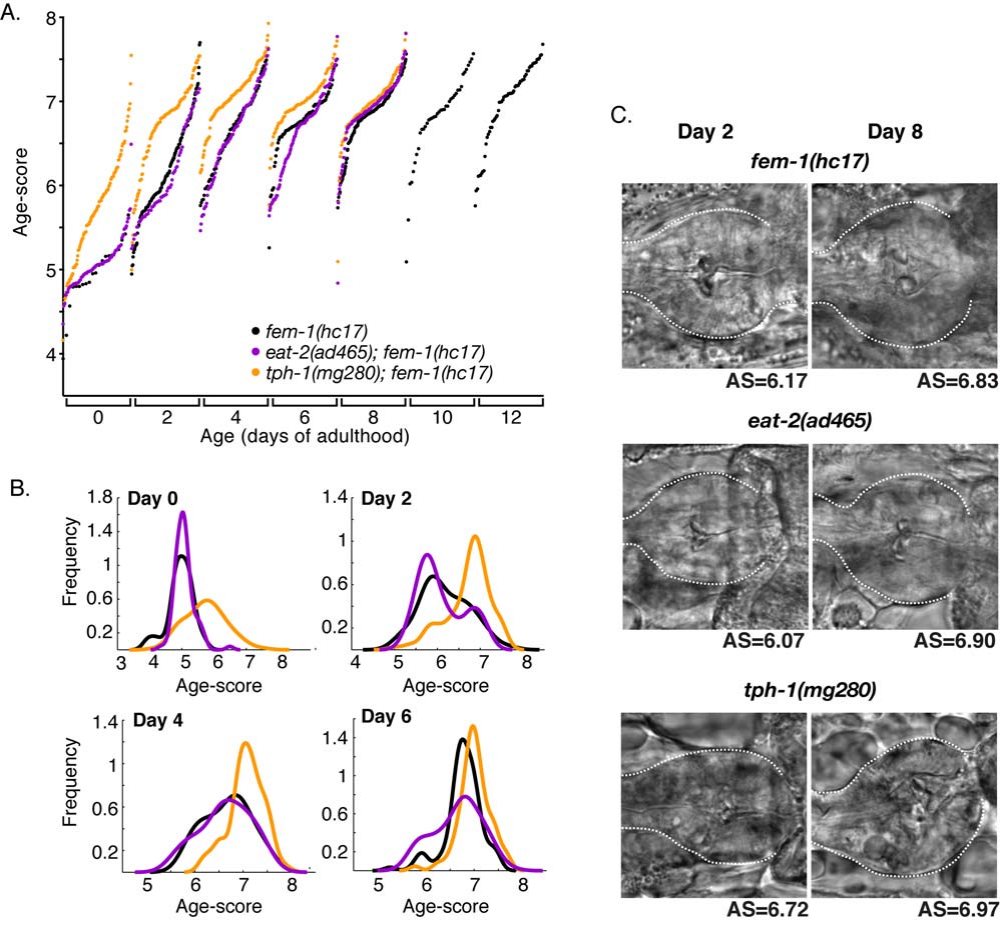
Image analysis revealed altered morphology states in slow-pumping *eat-2(ad465)* animals and serotonin-deficient *tph-1(mg280)* animals. (A) Age-scores assigned by a model trained on *fem-1(hc17)* days 0 to 12 for test images from normal and mutant animals were formatted the same way as Fig 3(B). The number of test images for each age class ranged between 68 and 117. (B) Probability distributions of *fem-1(hc17)*, *tph-1(mg280)*, and *eat-2(ad465)* age scores from days 0, 2, 4 and 6 from (A). A slight rightward shift is noted in day 6 *eat-2(ad465)* images. The images of *tph-1(mg280)* adults attained higher-than-normal age scores at all ages except day 6, possibly reflecting aberrant morphology. (C) Representative images of each strain at ages 2 and 8 were selected as having age scores (AS) near the population means. Lower left image of *tph-1(mg280*) day 2 pharynx reveals aberrant morphology. Lower right image of day 8 *tph-1(mg280)* shows structural deformation similar to that seen in day 8 *fem-1(h17)* and *eat-2(ad465)* animals.

We next examined whether the model could robustly detect pharynx morphology differences between normal (*fem-1(hc17)*) adults and mutants with defects in pharynx function. Early versions of the classifier software were able to distinguish a slow-aging appearance in pharynx images from long-lived strains carrying mutations in the *daf-2* gene, encoding an insulin-receptor like protein (Fig. S1) [14]. Since delayed aging in the *daf-2*/insulin-like pathway mutants has been extensively documented, and likely involves hormonal control of aging in all tissues [1], [3], [15], [16], we turned our attention to mutants that more directly affect pharynx function. First, we examined pharynx morphology during aging of *eat-2(ad465)* animals, which lack function of a pharyngeal nicotinic acetylcholine receptor subunit [17]. The terminal bulb contraction rate in *eat-2(ad465)* adults is 75% slower than normal. This slow pump rate causes dietary restriction, prolonging lifespan in *eat-2(ad465)* adults by 30% [18]. Previous analysis had indicated that aging-associated pharynx structural decline was delayed in *eat-2(ad465)* animals [15]. This effect could be due to either dietary restriction's benefits or protective effects of slow pumping. To directly compare the previous analysis with the computational approach, we built a model trained on pharynx images from normal day 2 and 8 animals, and used it to score pharynx images from day 2 and 8 *eat-2(ad465)* adults. This approach showed statistically significant lower scores between *eat-2(ad465)* and normal pharynx terminal bulbs at adult day 8 (*p* = 0.014), indicating that the computational approach was consistent with human-based scoring in a side-by-side comparison (Fig. 4). However, scoring *eat-2(ad465)* pharynx images from all available ages (days 0–8) using the complete *fem-1(hc17)* model (days 0–12) showed that *eat-2(ad465)* images scored significantly younger only at day 6 (p = 0.04, one-tailed t-test), with a small downward trend at day 2 (Fig. 5). These results demonstrate that the computational model allows construction of more complex visual assessments than human-based scoring when quantifying the relative differences in morphology between these strains. Further, the model's results provide a more quantitative assessment of the relatively modest effect of the *eat-2(ad465)* mutation on pharynx muscle aging.

### Serotonin deficiency alters pharynx development but has neutral effects on structural transitions during aging

We next examined pharynx images from *tph-1(mg280)* animals, which are unable to synthesize serotonin [19]. The deficiency in endogenous serotonin causes a 40% reduction in pharynx pump rate. First, we used a limited model trained on normal day 2 and 8 adults. Surprisingly, this model predicted high age scores for *tph-1(mg280)* images at day 2 (not shown). This analysis was extended to examine *tph-1(mg280)* day 0–8 images with the complete model trained on day 0–12 *fem-1(hc17)*. The complete model also predicted high age scores for *tph-1(mg280)* images (Fig. 5A, B). Further analysis of these results indicated that *tph-1(mg280)* adults entered the State II and III morphologies at younger ages than normal adults. In particular, the distribution of age scores for day 2 *tph-1(mg280)* animals was indistinguishable from those which define the State II morphology state in normal animals between days 4–10 (*p*≥0.05). Furthermore, images from day 4–8 *tph-1(mg280)* animals most resembled day 12 normal animals in State III (*p* = 0.1, 0.6, 0.9 for *tph-1(mg280)* days 4, 6, and 8, respectively). These relationships were not dependent on the specific images used for training and testing, as similar results were obtained from a model trained on *tph-1(mg280)* images and tested with images from normal adults. We interpreted these findings to indicate that *tph-1(mg280)* and normal animals populated distinct morphology states during early adulthood, but converged onto shared states at older ages, though with *tph-1(mg280)* arriving at these states ahead of normal.

The older age scores for day 0 and 2 *tph-1(mg280)* images could reflect accelerated aging in this mutant, or simply an aberrant morphology unrelated to normal aging. A visual inspection of representative images chosen from these groups was performed to examine the basis for the computed age scores (Fig. 5C). In day 2 adults, we observed that *tph-1(mg280)* pharynx terminal bulbs appeared unusually shaped and less rounded than in *fem-1(hc17)* and *eat2(ad465)* adults (Fig. 5C). The aberrant *tph-1(mg280)* morphologies may be a factor contributing to the computed older age scores, although they were visually distinguishable from normal old-age morphologies. In day 8 *tph-1(mg280)* pharynx images, morphological deterioration similar to that of normal aging was evident, accounting for the convergence in age-scores with *fem-1(hc17)* controls. An altered pharynx morphology has not been previously described for *tph-1(mg280)* animals. However, prior analysis using human subjects performing blinded comparisons of day 2 and 8 *tph-1(mg280)* pharynx terminal bulb images revealed a trend towards scoring day 2 and 8 images similarly, although this did not attain statistical significance, possibly due to small sample size [15]. Together, these findings demonstrate that *tph-1(mg280)* animals exhibit a developmental defect that affects pharynx morphology in young adults. Furthermore, this altered morphology in young adulthood does not apparently protect against the normal pattern of morphological change with aging. Finally, this interpretation could be further explored with new models built to distinguish between the mutant and normal pharynx images independently of age.

### Longitudinal analysis identifies structural states correlated with future aging success

**Figure 6:**
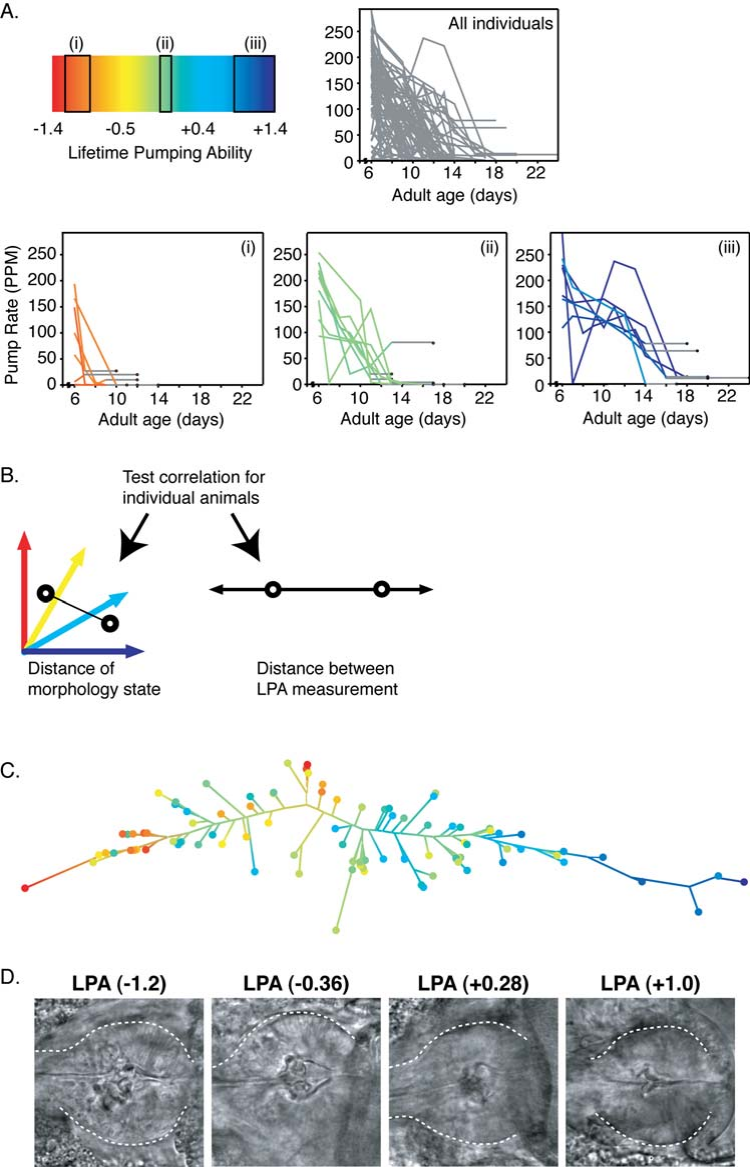
Image analysis identified morphological features correlated to pharynx function during aging. (A) Longitudinal pumping data are shown for individual animals sorted into low, medium, and high LPA ranges. Each animal's trace has a colored portion based on its LPA score that ends on the last day the individual was observed alive, and a horizontal grey portion that ends on the day the individual was observed dead. The light grey background lines depict pump rates measured for the entire population. Colors correspond to actual LPA value for each individual as depicted in the heatmap. The rectangles designate the actual LPA values for the individual data highlighted in the lower plots. These figures show that pump rates can have significant day-to-day variation, and that LPA values were positively correlated with pharynx function and healthspan after day 6. (B) Correlation between morphological differences and differences in future function was established by measuring distance between each pair of images in the LPA classifier's marginal probability space, and comparing it to the corresponding difference in LPA values. (C) Dendrogram representing image similarity. 91 images are represented by branch endpoints with their relative positions predicted by the model. Endpoint colors indicate the LPA value for each image; branch colors are determined from the relative contributions of neighboring endpoints. This dendrogram shows that the LPA classifier was able to correctly order images with respect to their measured LPA values, from low (red and orange) to high (blue), as designated in part (A). (D) Representative images show a progression from high to low levels of structural deterioration that corresponds to their measured LPA values.

An important issue for understanding the biology of aging is defining whether changes associated with early aging can correlate reliably to future aging success. We addressed this question for the *C. elegans* pharynx by searching for longitudinal correlations between mid-life morphology and future functional decline. First, we measured pharynx pump rates and collected pharynx images from day 6 adults, an age where the population exhibits a wide variation in pump rate due to heterogeneous functional declines [15]. It was also felt that day 6 animals would be better able to tolerate the imaging procedure than older animals (see Methods). Following image acquisition, animals were recovered and pharynx pump rates were determined every few days until death. As expected, average pharynx pump rate in the population declined continuously during aging. However, we found that pump rates of individual animals exhibited substantial daily variation. This variation in day-to-day pump rates interfered with comparisons of pharynx function between animals. Therefore, we derived a composite measure of pharynx pump rate over lifetime, which we refer to as lifetime pumping ability (LPA). First, Z-scores were calculated that reflected individual deviation away from the population mean pump rate on each day. Then, an average was taken of each animal's Z-scores over the individual's lifetime to arrive at the LPA. Thus, the LPA provides a relative measure of pharynx function from day 6 onward while minimizing the effects of daily fluctuations. Comparisons of LPA with daily pump rate observations indicated that LPA was positively correlated with pharynx function after day 6 (Fig. 6A).

The day 6 pharynx images from the longitudinal data sets were first tested with the normal aging model trained on day 0–12 pharynx images (20 images/age). This model did not identify any significant correlations between age-score and either LPA or lifespan. However, it was possible that the day 6 images contained features related to function that were ignored by the aging model. To more directly search for morphological correlations to LPA, a model was trained on the entire longitudinal image set segregated into quartiles by their associated LPA scores. Because separate test images were not used to assess this model, we tested the model's significance by determining the correlation between the self-imposed class order derived from image features and the observed LPA scores (Fig. 6B). Using this approach, the Pearson correlation between inter-image distances and corresponding differences in LPA scores was 0.40 compared to 0.18±0.08 for 20 negative controls using randomized image∶LPA assignments (z-test *p* = 0.0017). Thus, image features apparent at day 6 could be used to differentiate between individual pharynx images on the basis of future pharynx function over the remaining lifespan. Comparison of the features used in the LPA model to those used in the age-prediction model revealed very little overlap: the angle between the global feature weight vectors was 89.9 degrees, indicating the models' weighted feature subspaces were essentially orthogonal. Additionally, of the 43 image features used by the LPA model and the 28 used by the aging model, only three were common to both. Thus, the morphological features related to pharynx age at mid-life differ from those related to pharynx function.

A dendrogram was constructed from the inter-image distances produced by the model built from the longitudinal image set (Fig. 6C). This figure demonstrates that the model was able to order the images by LPA. This order was generated from image content alone because the model was trained with unordered classes. Visual inspection of selected images revealed more extensive structural deterioration in the terminal bulbs of animals with lower LPA than in those with higher LPA values (Fig. 6D). The computational and visual observations together suggest the existence of function-related morphology traits that describe the continuum of LPA values. These data also support a relationship between pharynx morphology and aging success of this tissue.

## Discussion

Little is known about mechanisms that promote tissue deterioration during aging. Current thinking holds that tissue structural decline may result from mitochondrial dysfunction, wear and tear, or reduced regenerative capacity at older age [15]. One approach to identifying pathways potentially involved in aging-related declines has been comparing global gene expression in young and aged samples. Transcriptional profiles of aging have been described for whole *C. elegans* nematodes and for tissues from *Drosophila melanogaster* fruitflies, mice and humans [20]–[26]. Together, these studies revealed conserved declines in expression of genes encoding components of the mitochondrial respiratory chain, suggesting some shared aspects of tissue aging among these diverse organisms [26], [27]. Interestingly, transcriptional changes associated with aging in both *C. elegans* and *Drosophila* were found to occur at relatively young ages, between young adulthood and mid-life [23], [27]. This is in accordance with our finding of a correlation between mid-life pharynx morphology and future functional decline, as measured by LPA. Thus, both transcriptional and morphological measures provide evidence for early- to mid-life effects of processes relevant for aging.

Muscles provide reliable and generally applicable biomarkers for aging. In human muscle, transcriptional changes associated with chronological age correlate with physiological age, as defined by the ratio of Type I to Type II muscle fiber size fibers [26]. Longitudinal studies in elderly humans correlated muscle weakness with earlier death [28]–[30]. In *C. elegans*, functional declines in locomotory and feeding muscles correlate with lifespan [1], [16], [31]. In this study, we found that structural aspects of muscle aging were correlated with future functional decline within a tissue. Interestingly, features relating to adult age were distinct from those relating to function, as evidenced by the finding that the age-defined model was unable to predict future functional decline. It remains possible that certain structural changes are associated with function, but these must occur after day 6, the age at which pharynx images were collected in our longitudinal experiment. These findings imply that a broad range of independent processes affect morphology in adult animals, some of which govern age-related structure, and some of which govern functional decline.

One notable result from our analysis was the presence of distinct pharynx morphology states during aging in wildtype animals, as opposed to a gradual progression of morphological change. Two observations indicate that the morphology stages of aging in the *C. elegans* pharynx are present in the biological samples rather than artifacts of this type of analysis. First, the number and timing of the morphological states was not completely defined by the age classes used during training. This is most clearly illustrated by the partial occupancy of the mid-life state (State II) at a range of ages. Furthermore, similar states were identified in two distinct pharynx regions, and state transitions appeared to be synchronized in these regions within individual animals. Thus, our analysis revealed that structural aging occurs synchronously within a tissue or organ. Synchronous aging-related changes were also observed at the level of global gene expression in human kidney regions [25].

The existence of distinct structural states disputes the theory that aging decline is completely random. Rather, we posit that these morphological states represent stable structures arising through the action of multiple pathways. We feel they are not likely to represent dominant structures formed through a single pathway. First, the morphological states are heterogeneous, suggesting that they are not achieved through a single pathway. Second, analysis of *tph-1(mg280)* animals indicated that, although these animals entered adulthood with an aberrant pharynx morphology, they nevertheless reached the stable mid-life state we termed State II. This offers evidence that State II morphologies can be achieved from different starting points, such as through the action of alternate pathways to a shared outcome. In light of these possibilities, it will be interesting to determine whether morphological states and their transitions are tissue-intrinsic, or are widely detected in a number of the body's tissues.

The pattern recognition approach to image analysis presented here identified correlations between a range of morphologies and continuous experimental variables (age and LPA score), and discovered stable distinct morphological states spanning training classes. The complex nature of the images used in this study prohibited traditional image analysis approaches that rely on explicit object identification within each image. Rather, the methods we developed employed a general-purpose bank of image descriptors that measured global image content without assumptions about the imaging modality or object identification. Although the features used in this method can be quite abstract (e.g. Fourier textures), visual inspection of randomly-selected representative images could often confirm identified trends, even when these trends were not identified *a priori* [15]. The general nature of this whole-image analysis method may make it applicable to many other previously intractable quantification problems in cell biology and tissue morphometry, including dose-response relationships, image-based high-throughput screens, and medical diagnostics applications.

The difficulty of correlating the visual cues used in machine vision with those used by humans is a limitation of this approach to image analysis. As early as 1690, in his “Essay concerning humane understanding”, John Locke observed “If that most instructive of our senses, seeing, were in any man a thousand or a hundred thousand times more acute…He would come nearer to the discovery of the texture and motion of the minute parts of corporeal things…But he would be in a quite different world from other people: nothing would appear the same to him and others…So that I doubt, whether he and the rest of men could discourse concerning the objects of sight” [32]. It is true that using this approach precludes a “discourse concerning the objects of sight”, and by extension the underlying mechanism of age-related muscle degeneration. However, the findings in this work–specifically the discovery of preferred morphological states in adult aging-demonstrate that it is not always necessary to have a complete understanding of the underlying perceptual model in order to use it to observe new phenomena. These techniques are not meant to supplant model-based image analysis based on prior understanding, but rather enable the analysis of images where no visual model exists.

The software developed here is open-source and is freely available as part of the OME project (Open Microscopy Environment, http://openmicroscopy.org).

## Materials and Methods

### *C. elegans* strains and methods

*C. elegans* strains used in this work were BA17, *fem-1(hc17)*; CY302, *fem-1(hc17)*; *tph-1(mg280)* and CY303, *fem-1(hc17)*; *eat-2(ad465)*. Some strains were provided by the *Caenorhabditis* Genetics Center at the University of Minnesota. Construction of CY302 and CY303 was as follows. Heterozygous *fem-1(hc17)* males were obtained by crossing wildtype (N2, Bristol) males to BA17 fertile hermaphrodites and isolating F1 cross-progeny males, which were mated, to young adult hermaphrodites carrying either the *tph-1(mg280)* or *eat-2(ad465)* mutations (strains GR1321 or DA465, respectively). Independent F3 progeny were clonally derived from F2 progeny of the second cross and screened for the presence of the *eat-2(ad465)* mutation (by measuring pharynx pump rate) or the *tph-1(mg280)* mutation (by PCR-amplification using gene-specific primers). After homozygous populations for these mutations were identified, progeny were screened for *fem-1(hc17)* homozygosity as 100% sterile adults at 25°C. We confirmed that CY303 had extended lifespan as previously reported [18]. We also tested the longevity of CY302 and found that it was identical to that of the *fem-1(hc17)* parental strain (not shown).

All animals were cultivated following standard protocols on NGM agar plates with the *Escherichia coli* strain, OP50, as a food source [33]. Age-synchronized populations were obtained by allowing fertile adult hermaphrodites to lay eggs on NGM medium with bacterial food for several hours at 15°C. The eggs were shifted to 25.5°C for 48 hours to complete development into spermless hermaphrodites. Day 0 of adulthood was defined as the day of the L4 to adult molt.

### Microscopy

For pharynx imaging, animals were transferred into buffered sodium azide (0.2%) positioned on a 2% agar pad on a standard microscope slide and then topped with a cover slip. The pharynx region of the anesthetized animals was imaged using a Hamamatsu ORCA-ER CCD digital camera mounted on a Nikon E800 upright microscope under an oil-immersion 60X objective using Nomarski optics. For each animal, the stage was rotated in order to maintain the same relative orientation in all images. All image and acquisition variables were held as constant as possible between sessions. OpenLab software was used to auto-calibrate exposure time, and to crop images. For longitudinal studies, pump rates in day 6 animals were measured on growth medium and individually mounted for pharynx imaging. After image collection, animals were recovered from the imaging medium and transferred to fresh growth medium in a drop of M9 buffer. Animals were maintained at 25°C on individual plates and their pump rates and survival were assessed until death, which was scored by the inability to move after gentle prodding with a platinum wire. Most animals ceased pharynx pumping several days before death. In control experiments, the imaging and recovery procedures were found to have negligible effects on adult lifespan (Fig. S2).

### Computational image analysis

20 training images for each age were randomly selected to build the model. A bank of algorithms converted each image into a feature-vector. Algorithms included polynomial decompositions, high contrast features, pixel statistics, and textures, which were computed from the raw image, transforms of the image, and transforms of transforms of the image [11], [34]. Each feature dimension was independently weighted for its ability to discriminate between training classes using its Fisher Discriminant score (FD) [12]. FD scores from two class groupings were used: 1) based on age classes, and 2) based on the slide the images were collected from. These slide-based FD scores identified features sensitive to variations in data collection, and were computed for each age group then averaged across age groups. The final weights used for analysis were the averaged slide-based FD scores subtracted from the age-based FD scores. No correction factor was applied to LPA-based classifiers, whose feature weights were determined solely from the FD scores of the LPA quartile classes. Dimensionality reduction of this feature weight vector, and scoring of individual images was performed as described in the text. The number of images used to test each model ranged from 20 per age group (Fig 2B), 21–177 per age group (Fig. 3B) and 68–117 per age and genotype (Fig. 5A).

Dendrograms were generated using the Fitch-Margoliash method implemented in the Phylip software package [35]. The dendrogram heat map was generated using a color-blending algorithm between the known values at each leaf node. For the age-based and LPA-based dendrograms, pair-wise distances were derived from the class-distance and class-probability sub-spaces, respectively.

### Synchronized classifiers for terminal bulb and corpus

The terminal bulb and corpus analysis was performed by randomly choosing 64 animals from each age group of days 2 to 8 for training, and the remaining 16–50 animals per age group for testing. Each animal was either a training or testing subject, and separate classifiers were trained on corpus or terminal bulb images from the same animals. Independent predictions for corpus and terminal bulb could be made for each animal in the test set.

## Supporting Information

Figure S1 Binary classification of pharynx images from daf-2(e1368) or (e1370) at day 15 and 20.
(1.98 MB TIF)

Figure S2 Survival of imaged and untreated (control) populations.
(0.84 MB TIF)
