# Supplementary figures and images for: Quantitative Image Analysis Reveals Distinct Structural Transitions during Aging in Caenorhabditis elegans Tissues
Source: PLoS One. 2008 Jul 30;3(7):e2821. doi: 10.1371/journal.pone.0002821 (PMC2483734; doi:10.1371/journal.pone.0002821)

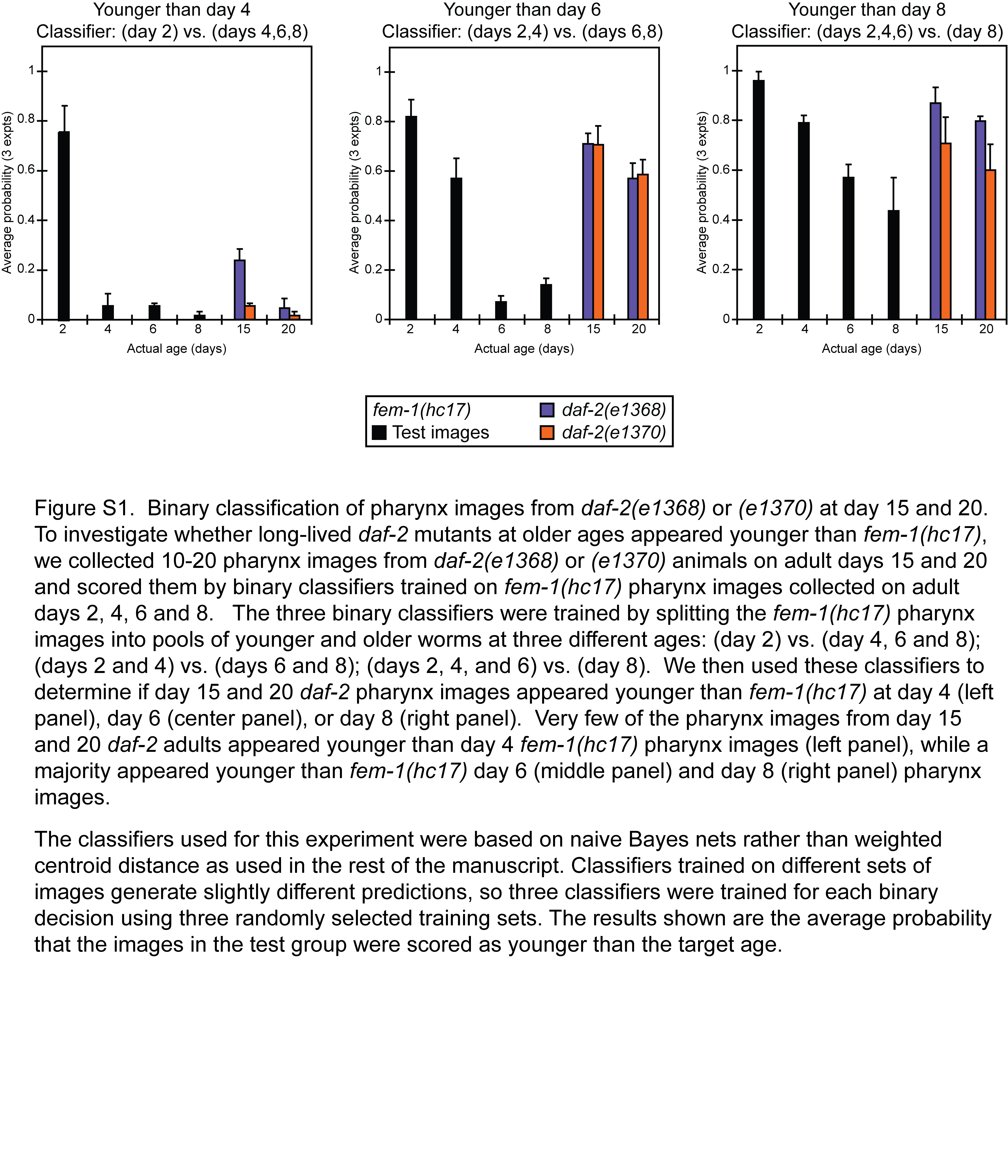

Supplement: Figure S1 — Binary classification of pharynx images from daf-2(e1368) or (e1370) at day 15 and 20. (1.98 MB TIF) [file pone.0002821.s001.tif]

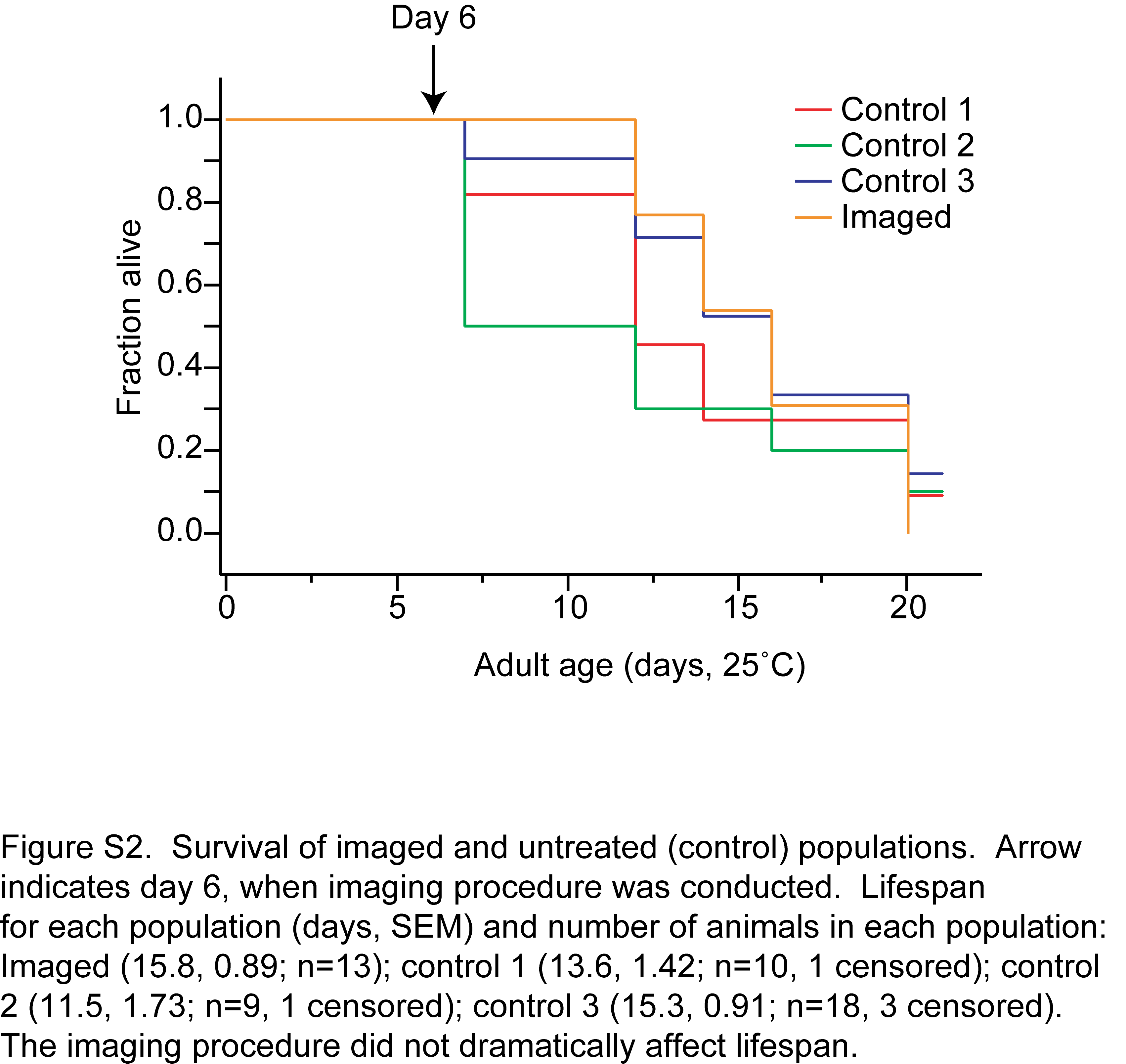

Supplement: Figure S2 — Survival of imaged and untreated (control) populations. (0.84 MB TIF) [file pone.0002821.s002.tif]
